# R21/Matrix-M™ malaria vaccine: a new tool to achieve WHO’s goal to eliminate malaria in 30 countries by 2030?

**DOI:** 10.1093/jtm/taad140

**Published:** 2023-11-11

**Authors:** Blaise Genton

**Affiliations:** Travel and Tropical Diseases Policlinic, Center for Primary Care and Public Health (Unisanté), University of Lausanne, Lausanne, Switzerland

**Keywords:** Malaria vaccine, R21/Matrix-M, RTS, S/ASO1, WHO recommendation, malaria, breakthrough

## Abstract

A potential breakthrough in the fight against malaria is the availability of a new promising tool, the R21/Matrix-M™ malaria vaccine that has shown an efficacy of 75% to protect young children against clinical malaria in different epidemiological settings. WHO recommends its deployment in addition to RTS,S/ASO1 and other effective interventions.

## The first malaria vaccine

In October 2021, a major step in the fight against malaria was achieved when WHO recommended RTS,S/AS01 (Mosquirix©; GlaxoSmithKline) malaria vaccine for the prevention of *Plasmodium falciparum malaria* in children 5–17 months living in regions with moderate to high transmission.^1^ In July 2022, WHO issued prequalification approval for this first malaria vaccine. The evidence behind the recommendation was that three doses of the vaccine achieved a protection of 56% against clinical malaria over 1 year. A booster dose administered at 18 months after completing the primary immunization conferred 36% vaccine efficacy against symptomatic malaria and 29% efficacy against severe malaria over 4 years of follow-up in the phase 3 trial.^2^ Since then, the Global Alliance for Vaccine Initiative (GAVI) board approved an initial investment of US$158 million (2022–25) to support rollout of the malaria vaccine in eligible countries.

The most recent update to the Malaria Vaccine Technology Roadmap highlights that, by 2030, one of the goals should be to licence malaria vaccines targeting *Plasmodium falciparum* that have a protective efficacy of at least 75% against clinical malaria for more than 2 years, in at-risk groups in malaria-endemic areas.^3^ This target has not been met by RTS,S/ASO1.

## R21/Matrix-M™ malaria vaccine, a potential game changer?

A significant progress to approach the target of Sustainable Development Goal 3 to end malaria in 2030 is the recent WHO recommendation to use R21/Matrix-M™ as a novel vaccine for malaria prevention, in addition to RTS,S/ASO1.^4^ This vaccine, developed at the University of Oxford, UK, and currently manufactured by the Serum Institute of India (Pune, India), has indeed proved safety and efficacy in Phase 1–3 trials conducted in UK and several sites in sub-Saharan Africa. R21/MATRIX-M™ is a pre-erythrocytic candidate malaria vaccine. It includes HBsAg fused to the C-terminus and central repeats of the circumsporozoite protein (CSP), which self-assemble into virus-like particles in yeast. Importantly, although this vaccine immunogen is similar to RTS,S, it does not have the excess HBsAg found in RTS,S and provides therefore a higher density of CSP epitopes on the particle surface, resulting in high levels of malaria-specific anti-Asn-Ala-Asn-Pro (NANP) antibodies.^5^ Following preclinical studies of R21 with multiple adjuvants, Matrix- M™ was selected for clinical development based on high immunogenicity. It is a saponin-based adjuvant produced by Novavax AB, Uppsala, Sweden, that stimulates both humoral and cellular immune responses.^6^ One advantage is that the adjuvant has been extensively tested and used as part of the Novavax Covid-19 vaccine (NVX-CoV2373).

As part of initial steps of the clinical development plan, a phase 2a trial conducted in UK showed R21/Matrix-M™ to be safe and provide protection of 82% after three doses of 10 μg, administered intramuscularly 4 weeks apart followed by a controlled human malaria infection (sporozoite challenge).^7^

Following an age de-escalation, dose selection, trial of R21/MATRIX-M™ in Kenyan adults, children and infants, which has shown a well-tolerated safety profile and potent immunogenicity,^8^ even in infants, a phase 2 trial in 450 children living in a malaria endemic area with high seasonal transmission was conducted in Burkina Faso. R21/MATRIX-M™—administered in 3 doses before the malaria season demonstrated an efficacy of 77% against clinical malaria over 1 year in the 5–17 months age group, thus reaching the WHO-specified efficacy goal of at least 75% in the target population of African children.^9^ The administration of a booster dose 12 months following the primary series of R21/MATRIX-M™ vaccinations showed that efficacy was maintained in the high-dose adjuvant group at 80% over 1 year, and 78% against multiple episodes of clinical malaria over 2 years after the primary three-dose regimen. In addition, R21/MATRIX-M™ had a favourable safety profile and induced high levels of malaria-specific anti-NANP antibodies.^10^

A phase 3 trial was initiated in 2021 at 5 sites in four sub-Saharan African countries (Burkina Faso, Mali, Tanzania and Kenya), two in high seasonal transmission areas and three in moderate to low perennial transmission areas, and 4800 children aged 5–36 months were enrolled to evaluate safety and efficacy of R21/MATRIX-M™ when given according to a 0-, 1- and 2-month schedule. Overall, vaccine efficacy after primary series was 75% (95% CI 71–79%) at the seasonal sites and 68% (61–74%) at the standard sites for time to first clinical malaria episode over 1 year of follow-up. At the seasonal sites, a booster dose administered at 12 months maintained an efficacy of 74% (70–77%) for time to first clinical malaria episode and 72% (68–75%) against multiple clinical malaria episodes for a surveillance period of 18 months. The concentration of antibodies against the conserved central NANP repeat sequence of CSP correlated strongly with vaccine efficacy. The results of the phase 3 are therefore fully consistent with those of the phase 2. Efficacy against severe malaria was 67% (−10–89%), but the number of cases was small and trial not powered to evaluate this outcome. Safety findings were reassuring with no vaccine-related serious adverse events and a well-tolerated safety profile. This included local pain at the injection site and fever as the only very common (>10%) adverse events.^11^

## How to integrate R21/MATRIX-M™ in the immunization WHO policy?

Given its excellent safety and efficacy across different epidemiological settings, R21/Matrix-M™ vaccine represents a promising tool for malaria control and elimination. This vaccine has shown efficacy for 2 years post-vaccination with a booster, with the potential for longer lasting protection due to its high immunogenicity. However, even if R21/Matrix-M™ has demonstrated suitability for implementation in regions with varying transmission intensities, it still lacks evaluation in areas with high perennial transmission. Since WHO is now recommending its deployment, which is excellent news, it will be important, albeit difficult, to accurately assess its efficacy in such contexts. Such an assessment is important since efficacy may be higher in sites with seasonal transmission because the vaccine is given just prior to the period of highest transmission, which renders the effect of waning protection over time less pronounced. Certainly, careful monitoring will be crucial to evaluate safety and impact of R21/Matrix-M™, since phase 3 trials do not provide all necessary information for a definite WHO policy. Detection of rare severe or serious adverse events, as well as impact on severe disease and mortality, can only be provided through phase 4 trials or large-scale deployment under careful monitoring with a strong pharmacovigilance system in place. Such an assessment is ongoing for RTS,S/ASO1 and preliminary results from the Malaria Vaccine Implementation Programme show a 29% reduction of hospitalisations with malaria parasitaemia and 7% reduction of deaths, which is reassuring.^12^ No such information is available for R21/Matrix-M™, which renders any policy decision weaker, or at least subject to fast revision during implementation. Because of the urgency to reverse the tide of increasing malaria cases and deaths since 2015, WHO released on 3 October 2023 the recommendation of deploying R21/Matrix-M™ malaria vaccine for malaria prevention in small children living in moderate to high endemicity areas. The simultaneous implementation of R21/Matrix-M™ and RTS,S/ASO1 will render head to head comparison difficult to conduct. Because Phase 3 trials have been conducted under different conditions (level of endemicity, populations, year), the SAGE and MPAG experts concluded that ‘there is no evidence to date showing one vaccine to perform better than the other. The choice of product to be used in a country should be based on programmatic characteristics, vaccine supply, and vaccine affordability’.^4^

The cost-effectiveness of R21/Matrix-M™ may still represent a comparative advantage since the price of the vaccine is announced at less than 5 dollars per injection. The low dose of R21 antigen used (5 μg) facilitates large-scale manufacturing. The Oxford team have a deal with Serum Institute of India to produce 100 million doses in 2024, and then up to 200 million doses annually. GAVI has estimated that steady-state requirement for the vaccine will exceed 80–100 million doses per year. Demand for vaccines to cover the target population of African children may thus outpace supply in the initial years of rollout. This highlights that there is definitely a room for both R21/Matrix-M™ and RTS,S/ASO1 to be deployed concurrently in the next years. The goal to access each child will only be met with concerted efforts.

## Malaria vaccines: relevance for travellers

Considerable efforts have been made in the development of malaria vaccines for travellers and soldiers, especially in the USA. The partial efficacy in adults in phase 1 and 2 trials in the USA and Mali of another malaria vaccine made of a whole sporozoite (PfSPZ from Sanaria) led some hope for the future besides R21/Matrix-M™ and RTS,S/ASO1.^13^ However, an efficacy below 90%, which is the case for the few vaccines that have shown efficacy in phase 2 or 3 trials, will never beat that of chemoprophylaxis. R21/Matrix-M™ could be an option because of its good efficacy and potential longer protection. However, the risk for non-immune individuals to develop severe malaria despite vaccination will probably still be too high to recommend it widely. It is therefore unlikely that vaccination will replace chemoprevention for travellers in highly endemic areas in the near future. Vaccination could be a useful tool for those who refuse chemoprevention, as an adjunct to standby emergency treatment. One might also consider vaccination for long-term travellers who are more prone to stop chemoprevention, but booster will need to be considered after one year, which may pose a problem for purchasing. Malaria vaccination is not an option at this stage for travellers or maybe a possible alternative for special cases with appropriate advice.

## Conclusion

RTS,S/ASO1 laid the foundation for malaria vaccine development and remains a pivotal step in the battle against malaria. The R21/Matrix-M™ vaccine, with its remarkable efficacy, potential durability and low price, is a welcome additional tool to fight malaria. Its production in an endemic country is also a significant advance in ownership. To date, it has already been licensed for use in Ghana, Nigeria and Burkina Faso. Ultimately, the success of either vaccine hinges on global collaboration, investment and a commitment to ensure equitable access. Moreover, the journey to eliminate malaria demands the thoughtful integration of these vaccines with other tools (e.g. Seasonal Malaria Chemoprevention) to achieve our shared goal of zero malaria.

## References

[1] https://www.who.int/news/item/06-10-2021-who-recommends-groundbreaking-malaria-vaccine-for-children-at-risk (10 October 2023, date last accessed).

[2] RTS,S Clinical Trials Partnership . Efficacy and safety of RTS,S/AS01 malaria vaccine with or without a booster dose in infants and children in Africa: final results of a phase 3, individually randomised, controlled trial. Lancet 2015; 386:31–45. 25913272 10.1016/S0140-6736(15)60721-8 PMC5626001

[3] Malaria Vaccine Funders Group . Malaria Vaccine Technology Roadmap. November, 2013. https://www.who.int/publications/m/item/malaria-vaccine-technology-roadmap (10 October 2023, date last accessed).

[4] https://www.who.int/news/item/02-10-2023-who-recommends-r21-matrix-m-vaccine-for-malaria-prevention-in-updated-advice-on-immunization (10 October 2023, date last accessed).

[5] Collins KA, Snaith R, Cottingham MG et al. Enhancing protective immunity to malaria with a highly immunogenic virus-like particle vaccine. Sci Rep 2017; 7:46621. 28422178 10.1038/srep46621 PMC5395940

[6] Datoo MS, Madhavan M, Bellamy D et al. Looking ahead in malaria: R21/Matrix-M™, an exciting new vaccine candidate. Am J Trop Med Hyg 2020; 103:469.

[7] Venkatraman N, Bowyer G, Edwards N et al. High level efficacy in humans of a next-generation Plasmodium falciparum anti-sporozoite vaccine: R21 in Matrix-M (TM) adjuvant. Am J Trop Med Hyg 2017; 97:594.

[8] Njau IW, Datoo MS, Sang S et al. A Phase Ib, open-label, age de-escalation, dose escalation study to evaluate the safety and tolerability of different doses of a candidate malaria vaccine adjuvanted R21 (R21/MM) in adults, young children and infants in Kilifi, Kenya. Am J Trop Med Hyg 2020; 103:226.

[9] Datoo MS, Natama HM, Somé A et al. Efficacy of a low-dose candidate malaria vaccine, R21 in adjuvant Matrix-M, with seasonal administration to children in Burkina Faso: a randomised controlled trial. Lancet 2021; 397:1809–18. 33964223 10.1016/S0140-6736(21)00943-0 PMC8121760

[10] Datoo MS, Natama HM, Somé A et al. Efficacy and immunogenicity of R21/Matrix-M™ vaccine against clinical malaria after 2 years' follow-up in children in Burkina Faso: a phase 1/2b randomised controlled trial. Lancet Infect Dis 2022; 22:1728–36. 36087586 10.1016/S1473-3099(22)00442-X

[11] Datoo M, Dicko A, Tinto H et al. A phase III randomised controlled trial evaluating the malaria vaccine 1 candidate R21/Matrix-M™™ in African children. Lancet. https://papers.ssrn.com/sol3/papers.cfm?abstract_id=4584076 (10 October 2023, date last accessed).

[12] Malaria Vaccine: WHO position paper. Weekly epidemiological record. 2022; 97:61–80.

[13] Sissoko MS, Healy SA, Katile A et al. Safety and efficacy of PfSPZ vaccine against Plasmodium falciparum via direct venous inoculation in healthy malaria- exposed adults in Mali: a randomised, double-blind phase 1 trial. Lancet Infect Dis 2017; 17:498–509. 28216244 10.1016/S1473-3099(17)30104-4 PMC6803168

